# The Standardisation of Digitalis Preparations

**Published:** 1907-03-02

**Authors:** 


### THE STANDARDISATION OF DIGITALIS PREPARATIONS.

It is probable that, of all cardiac tonics, digitalis is the best we have, and it is the most widely used. Many lives have been saved, or at least prolonged, by it. It is of vital importance that the drug should contain its full quantity of active principles. Nevertheless it has been shown conclusively, by Dr. W. E. Dixon and others, that a great deal of the tincture of digitalis as obtained upon the market is extremely weak, and some of it is even quite inert. This is not due to any fraud or error on the part of those who manufacture the tincture, which may, indeed, have been made in the orthodox way from apparently the best foxglove leaves, and yet the result may be little more than a coloured solution of alcohol. This apparently depends upon the fact that there is very great variation in the amount of active principle in different specimens of foxglove leaves, and until recently there has been no means of distinguishing the active leaves from the inert. No doubt the differences depend upon such conditions as the time of year at which the leaves are gathered, or the nature of the soil upon which the plants have grown—factors which can be controlled; but they also seem to depend upon certain uncontrollable factors, such as the amount of sunshine there has been whilst the plant was growing, the amount of moisture in the air, the amount of moisture in the soil, the temperature, and so on. Leaves gathered from similar plants, in the same locality, at the same season, but in different years may vary widely in their respective therapeutic values. It is possible that those who have used tincture of digitalis in full doses without benefit, or even perhaps with apparent ill-effects, have been supplied with a deficient or inert sample, so that the patients have been getting either very little real digitalis or even none at all. The chemist who supplies the ordinary tincture cannot guarantee its efficacy. However well the tincture may have been prepared, and however fresh it may be, the chemist, in the case of the B.P. tincture, can only guarantee that it has been prepared from apparently good leaves according to the official formula. Neither the chemist nor the doctor can tell beforehand that the required active principle is present.

This is a very serious matter. In many of the cases in which the drug is needed, the conditions are too urgent to allow time for experimenting on the patient to find out whether this or that sample of digitalis is the more active. The activity of any given sample should be known with certainty beforehand. In other words, the preparations of digitalis, to be ideal, should be standardised.

At first sight such standardisation sounds easy. There are many drugs which are standardised already; for example, nux vomica and opium. These and others are standardised by chemical analysis, the amount of active principle being directly estimated. Why not standardise digitalis in the same way? Attempts to do so have been made repeatedly, and many so-called active principles, such as digitalin, digitoxin, and so on, have been prepared. These, however, are mostly of much less benefit to patients than are the liquid preparations of the crude drug, and the chief cause of this is the fact that the active principle of foxglove is not an alkaloid like strychnine and morphine, but a *glucoside* which, like other glucosides, *decomposes when attempts are made to dry it.* In consequence of this it has been found practically impossible to standardise digitalis by any chemical means. The question arises: Is there no other method of standardising the drug? The answer is: Yes, there is; there is a *physiological* method. A physiological method of standardising therapeutic preparations is by no means new. It is well known in the case of antidiphtheritic serum, where guinea-pigs are used for the purpose. Dr. Dixon has devised a somewhat similar method for digitalis, using pithed (*i.e.* not living) frogs as the physiological medium. He has determined what is the minimum quantity of a standard tincture or infusion of digitalis that, injected into an average frog, will cause the heart to stop beating. Using this as a basis of comparison, he obtained samples of digitalis preparations from various sources, and determined what was the least dose of each that would similarly stop the heart. He frequently found that more than twice the standard minimum dose was needed; in other words, the tinctures as bought contained less than half the active principle they should have contained. It will be obvious that the dose should be at least twice as large with these as with the standard tincture—*i.e.*, at least 30 minims would have to be administered to produce the physiological effect expected from 15 minims of the standard preparation. Expressed in another way, the dosage cannot be arbitrarily laid down as a uniform quantity for all tinctures of digitalis, but rather the dose will vary with each brew of tincture. After it has been determined physiologically in frogs, the dose can be stated on each bottle, to the effect, for example, that "to produce the physiological effect of a standard 15 minim dose it is necessary to give 30 minims (or 20, 25, 35, 15, and so on, as the case may be) of this particular tincture."

There are many firms who now standardise their digitalis preparations in this way. The cost is, of course, slightly more for the standardised than for unstandardised medicines; but seeing how important it is to have efficient preparations, the fact that physiologically standardised digitalis is obtainable cannot be too widely known to the medical profession.

It may be added that digitalis is not the only glucoside drug which is now standardised physiologically. Strophanthus, squill, cannabis indica, and ergot, may all be obtained standardised in a similar way. None of these can be standardised by chemical analysis for the reasons stated above, but it is possible to determine the relative physiological strengths of different preparations of each of them; and this is particularly important in the case of ergot, so many samples of which are practically inert.

A point in regard to the vomiting which so often follows the administration of tincture of digitalis is worthy of note. Tincture of digitalis is frequently prescribed in a mixture along with nux vomica, or strychnine, or caffeine, and so on. If vomiting should follow, prescribe the tincture of digitalis quite by itself in pure water, and vomiting will almost certainly cease, even when full doses are given. It is when it is given simultaneously with other drugs that digitalis is liable to cause vomiting.

It is also worthy of note that digitalis does not act rapidly upon the heart. It takes time to develop its full effect. One of the best indications of improvement in a failing heart is an increase in the amount of urine passed. It will usually be found that the urine does not materially increase in quantity until the third or fourth day after the administration of digitalis has been begun. In other words, it takes from three to four days before the digitalis begins really to relieve the cardiac failure. This being so, it is obvious that digitalis by itself is not the drug for an acute cardiac emergency, and it helps to explain why digitalis acts so much better in failure from mechanical lesions of the valves than it does in cases of, for example, acute pericarditis, or acute fevers such as pneumonia or typhoid fever.

